# Efficacy and Safety of Qinghao Biejia Decoction in the Treatment of Systemic Lupus Erythematosus: A Systematic Review and Meta-Analysis

**DOI:** 10.3389/fphar.2021.669269

**Published:** 2021-08-06

**Authors:** Xiaobo Li, Zhouqing He, Li Ru, Yueming Yuan, Zheng Yuan, Pengfei Chen, Zhiyong Xu, Qi Wang, Jianping Song, Qin Xu

**Affiliations:** ^1^ Artemisinin Research Center, Guangzhou University of Chinese Medicine, Guangzhou, China; ^2^ Sci-tech Industrial Park, Guangzhou University of Chinese Medicine, Guangzhou, China; ^3^ The First Affiliated Hospital of Guangzhou University of Chinese Medicine, Guangzhou, China

**Keywords:** Qinghao Biejia decoction, systemic lupus erythematosus, meta-analysis, efficacy, safety

## Abstract

**Objective:** This meta-analysis aimed to systematically assess the therapeutic efficacy and safety of Qinghao Biejia decoction combined with conventional chemical medicine in patients with systemic lupus erythematosus (SLE), and to provide reference for clinical medication.

**Methods:** Multiple databases were retrieved by computer for randomized controlled trials (RCTs) of treating SLE with Qinghao Biejia decoction combining chemical medicine, from the establishment of the database to January 2021. Study screening, data collection, and quality assessment were performed independently by two reviewers. RevMan 5.4 and Stata 15.1 software were used for Meta-analysis.

**Results:** Nine eligible studies, involving 655 SLE participants, were included. Meta-analysis showed that the additional use of Qinghao Biejia decoction superior to chemical medicine alone in people with SLE in improving the overall response rate (RR = 1.30, 95% CI [1.19, 1.41], *p* < 0.00001, heterogeneity *p* = 0.61, *I* ^*2*^ = 0%), and can decrease SLE Disease Activity Index (SLEDAI) and TCM symptom scores, improve immunological indexes (C3, C4, IgG, IgA, IFN-γ, IL-4, Th1/Th2), reduce the occurrence of adverse events in treatment (*P* ≤ 0.05).

**Conclusion:** Based on this meta-analysis, the additional use of Qinghao Biejia decoction has more advantages in the treatment of SLE than conventional chemical medication alone, which could enhance the efficacy and reduce adverse reactions, and is worthy of clinical promotion. However, more and higher quality RCTs are still need to confirm our findings.

## Introduction

Systemic lupus erythematosus (SLE) is an autoimmune disease with a complex pathogenesis, which can be involved in all systems of the human body, and can damage multiple target organs. Fever, rash, and arthritis are the classic initial symptoms. Abrupt onset with target-organ involvement is also common, such as hematologic findings (thrombocytopenia, leukopenia, lymphopenia, or anemia), renal findings (hematuria, proteinuria, cellular casts, or elevated serum creatinine level), respiratory symptoms (cough, dyspnea, hemoptysis, or pleuritic pain), central nervous system signs (headache, photophobia, or focal neurologic deficits) (Kiriakidou and Ching, 2020). The morbidity of SLE is very high, mostly in young women. Globally, estimates of SLE prevalence in adults range from 30 to 150 per 100,000, and incidence ranges from 2.2 to 23.1 per 100,000 per year (Durcan et al., 2019). Approximately 1.3 million people in China suffer from SLE, with an average prevalence of 0.0975–0.376% (Carter et al., 2016).

There is no cure for SLE, but it can be managed with medications. At present, medicine treatment predominantly involve immunomodulation and immunosuppression, such as glucocorticoids, cyclosporine, azathioprine, cyclophosphamide, hydroxychloroquine (Durcan et al., 2019; Piga and Arnaud, 2021). However, these drugs have many adverse effects, may cause or aggravate the impair of other organs and infections with weakened immune function. Therefore, it is urgent to develop more effective drugs and optimize the use of those currently available. Current research shows that traditional Chinese medicine treatment or combine with chemical medicine can reduce the adverse effects of chemical drugs and dosage of steroid, improve the symptoms, immune function of SLE patients and quality of life, the body’s self-regulation ability can be gradually enhanced, while reducing complications and consolidating clinical efficacy (Yang et al., 2016; Chen et al., 2019; Fu et al., 2020).

Qinghao Biejia decoction is one of the classic prescription in ancient China, which is composed of Qinghao (*Artemisia annua* L.), Biejia (*Trionyx sinensis* Wiegmann), Zhimu (*Anemarrhena asphodeloides* Bunge), Shengdihuang (*Rehmannia glutinosa* (Gaertn.) DC.), and Mudanpi (*Paeonia × suffruticosa* Andrews). It has the effects of antipyretic, anti-inflammatory, improving immunity, sedation, anti-pathogenic microorganisms, etc., and can be used in the clinical treatment of cancer fever, various inflammation, postoperative fever, rheumatic immune disease, blood disease and climacteric syndrome (Yu, 2012; Wang et al., 2016; Lin and Li, 2017; Xia and Chen, 2019).

A number of clinical studies have reported that Qinghao Biejia decoction or its modified has a reliable effect in the treatment of SLE, which can restore body temperature, relieve joint pain, reduce erythema, and eliminate edema, etc (Zhong and Li, 2004; Liu and Wang, 2006; Zhong et al., 2010; Zang and Zhang, 2015). When combined with conventional chemical medicine, in addition to improving the clinical efficacy and controlling the disease, it also helps to reduce the dosage of steroid and the adverse effects (You, 2011; You, 2012; Gao et al., 2017; Cao, 2018; Bai and Zhao, 2019; Liu and Cao, 2019; Wan, 2019; Luo, 2020; Wang, 2020). However, due to few cases, different designs and efficacy indicators, there is a lack of convincing evidence to prove the effect of Qinghao Biejia decoction in treating SLE, which also affects its clinical promotion. So this study collected randomized controlled trials of Qinghao Biejia decoction combined with chemical medicine in the treatment of SLE to assess the efficacy and safety of Qinghao Biejia decoction for SLE through network meta-analysis.

## Methods

### Protocol Register

This systematic review and meta-analysis followed the methods of the Cochrane Handbook of Systematic Reviews and the guidelines from the Preferred Reporting Items for Systematic Reviews and Meta-Analyses (PRISMA) (Moher et al., 2009; Higgins and Green, 2011). The review protocol has been registered in PROSPERO (International Prospective Register of Systematic Reviews) before commencement (Registration number: CRD42021230393).

### Search Strategy

Medline, Embase, Google Scholar, China National Knowledge Infrastructure (CNKI), China Biology Medicine disc (CBM), Chinese Scientific Journals Database (VIP), Wanfang Database, etc. were retrieved with the retrieval words “Systemic lupus erythematosus”, “lupus erythematosus”, “SLE”, “Randomized controlled trials”, “RCT”, “Qinghao Biejia decoction”, “Qinghao”, “Biejia”, etc., in Chinese or English, and both use a combination of subject words and free words. The database was searched from their start date until January 2021.

### Eligibility Criteria

1) Type of study: only RCTs on Qinghao Biejia decoction treatment for SLE were eligible. The language of the trials to be included were restricted to Chinese and English.
2) Type of participants: adult patients who meet the diagnostic criteria of SLE.
3) Type of intervention: the control group was treated with chemical medicine alone, the treatment group was treated with Qinghao Biejia decoction combined with chemical medicine.
4) Type of results: The main outcomes included the overall response rate, SLE Disease Activity Index (SLEDAI) and TCM symptom scores. The additional outcomes were serum immunological indexes, such as complement (C3, C4), antiphospholipid antibody (IgG, IgA), cytokine (IFN-γ, IL-4, Th1/Th2). The safety was evaluated through adverse events.

### Exclusion Criteria

1) For duplicate publications, select the one with the most complete data.
2) Studies with obvious data errors or incomplete research index data.
3) The treatment group combined with other TCM therapies, such as acupuncture, massage, moxibustion and Qi Gong.

### Data Extraction and Quality Assessment

The titles and abstracts of the articles searched from the databases were read independently by two authors to select the eligible studies. Any different opinion of two authors were discussed to reach an agreement. Then two authors extracted data from the eligible trials independently by use of template which included the following: publication year, study design, age, number of cases, dosage and duration of intervention and comparison, outcomes, etc.

Both authors utilized the Cochrane Collaboration’s risk of bias tool to assess the risk of bias of the included studies. The quality of each study was graded as high, unclear, or low risk of bias with justifications (Higgins et al., 2011).

### Statistical Analysis

Statistical analysis was performed in the RevMan 5.4.1 software (Cochrane Collaboration, Oxford, United Kingdom) and Stata 15.1 (Stata Corp LLC, College Station, Texas, United States). Dichotomous data, such as the overall response rate and adverse events, was reported as relative ratio (RR), whereas continuous data such as SLEDAI and TCM symptom scores, laboratory indicators, were expressed as mean difference (MD) or standardized mean difference (SMD), and all the above were represented by effect value and 95% confidence interval (CI). The heterogeneity among trials was evaluated by *X* ^*2*^ and *I* ^*2*^ tests. If low heterogeneity (*p* > 0.1, *I* ^*2*^ < 50%), the fixed effect model was applied. If high heterogeneity (*p* ≤ 0.1, *I* ^*2*^ ≥ 50%), the random effect model was used. Sensitivity analysis was performed to evaluate the robustness of the results. Publication bias was examined by visual inspection of funnel plots for asymmetry and Egger’s test. The difference of *p* ≤ 0.05 in Meta analysis was considered statistically significant.

## Results

### Study Selection

**FIGURE 1:**
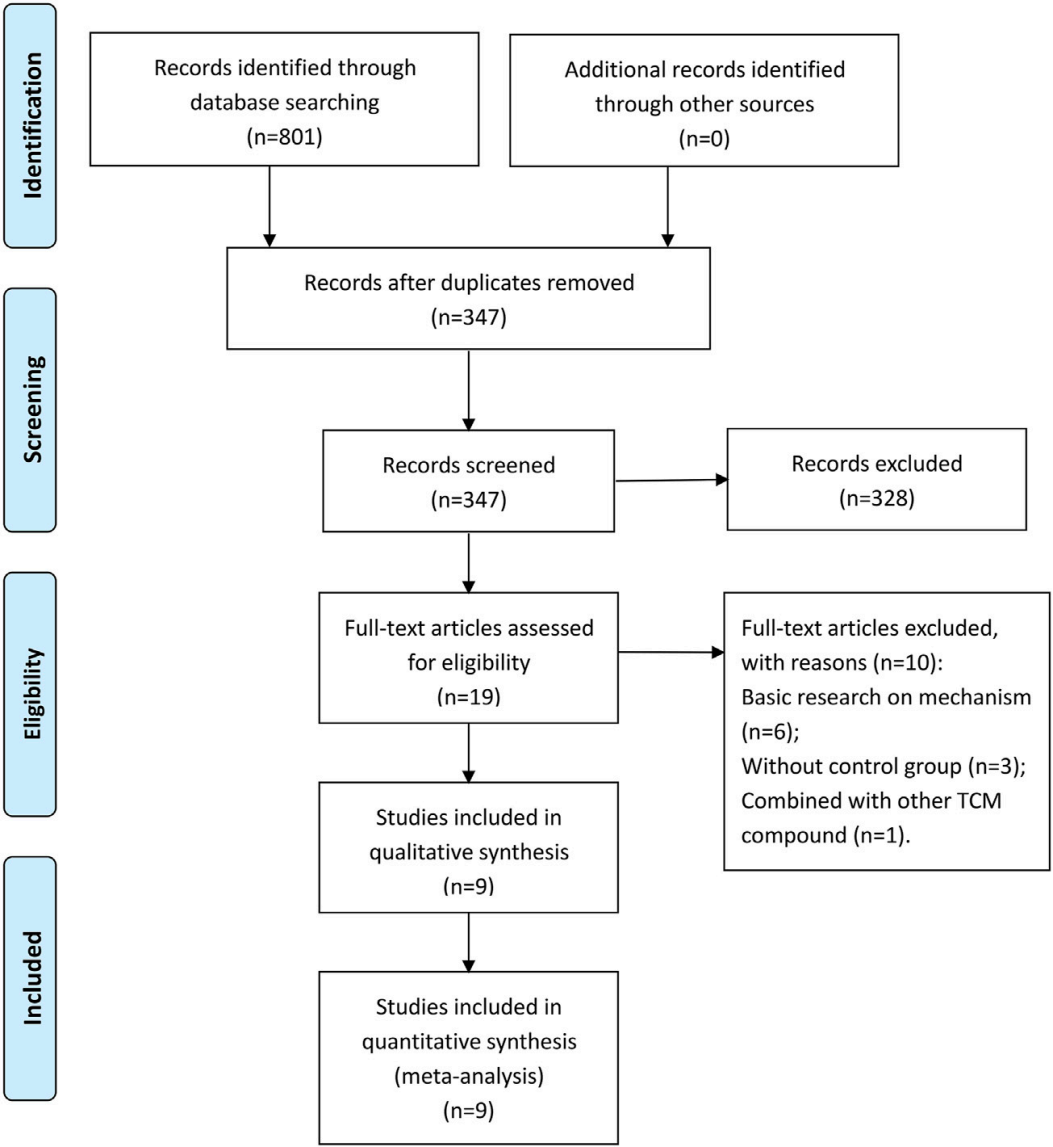
PRISMA flow diagram of screening process.

A total of 801 studies were retrieved from the databases, and 454 duplicate records were removed. In the remaining 347 articles, 328 articles were excluded by screening the titles and abstracts. By reading the full text, 10 studies were found inappropriate and hence deleted. Ultimately, nine eligible studies were included in this meta-analysis (You, 2011; You, 2012; Gao et al., 2017; Cao, 2018; Bai and Zhao, 2019; Liu and Cao, 2019; Wan, 2019; Luo, 2020; Wang, 2020). The study selection process is shown in Figure 1. All included studies were performed in China from 2009 to 2019 and published in Chinese-language medical journals from 2011 to 2020.

### Study Characteristics

The general characteristics of the included studies are presented in Table 1. A total of 655 adult participants with SLE were included in the nine eligible studies. The 323 cases in the control group were treated with conventional chemical medicines, including Hydroxychloroquine, prednison, cyclophosphamide. The 332 cases in the experimental group were treated with Qinghao Biejia decoction combined with chemical medicine in the control group. The treatment duration ranged from 12 weeks to 6 months.

**TABLE 1:** Basic characteristics of the included studies.

Included trial	Participants (M/F; age years)		Diagnostic criteria of SLE	Course of disease (month)		Intervention		Treatment duration	Outcome index
	Experimental	Control		Experimental	Control	Experimental	Control		
You (2011)	3/79; 20–50, 37.00 ± 7.77	2/71; 19–49, 35.82 ± 7.32	1997 ACR	6–18, 11.95 ± 2.78	6–16, 11.62 ± 2.16	QBD (one dose bid) plus CM	PED (1 mg/kg qd)	6 months	1) 2)
You (2012)	2/35; 20–40, 30.65 ± 5.03	1/36; 19–40, 32.22 ± 6.19	1997 ACR 2002 NMPA	6–18, 11.05 ± 2.79	6–16, 11.73 ± 2.99	QBD (one dose bid) plus CM	PED (1 mg/kg qd) plus CTX (0.5–1 g/m^2^ monthly)	12 weeks	1) 2) 3) 4) 5) 6) 7)
Gao 2017	3/27; 24–62, 38.31 ± 8.42	2/28; 21–58, 37.28 ± 7.10	2011 KTR 2002 NMPA	3–38, 17.06 ± 5.22	2–36, 16.47 ± 4.83	QBD (one dose bid) plus CM	HCQ (0.1 g bid) plus PED (40 mg qd)	3 months	1) 2) 3) 8) 9)
Cao (2018)	1/29; 21–56, 35.4 ± 7.1	4/26; 23–59, 33.8 ± 6.9	2002 NMPA	Not specified		QBD (one dose bid) plus CM	HCQ (0.2 g qd) plus CTX (0.6 or 0.8 g/m^2^ once every 3 months)	Not specified	1) 10) 11) 12)
Bai and Zhao (2019)	1/37; 22–60, 37.51 ± 7.25	2/36; 23–59, 37.43 ± 7.23	2003 RCMA 2002 NMPA	2–37, 16.02 ± 4.97	3–34, 15.97 ± 5.03	QBD (one dose bid) plus CM	HCQ (0.1 g bid) plus PED (40 mg qd)	3 months	1) 2) 3) 6) 13) 14) 15) 16)
Liu and Cao (2019)	11/7; 25–59, 34.2 ± 5.9	10/8; 24–58, 33.6 ± 6.5	1997 ACR 2002 NMPA	Not specified		QBD (one dose bid) plus CM	HCQ (0.2 g qd) plus CTX (0.6 or 0.8 g/m^2^ once every 90 days)	Not specified	1) 9) 11)
Wan (2019)	7/33; 23–59, average 40.82	6/34; 25–61, average 42.74	2002 NMPA	Not specified		QBD (one dose bid) plus CM	HCQ (0.1 g bid) plus PED (40 mg qd)	12 weeks	1) 9) 10) 11) 12)
Luo (2020)	4/16; 25–65, 43.7 ± 4.6	5/15; 26–66, 44.2 ± 4.3	SLE clinical diagnostic criteria	12–36, 15.6 ± 14.4	12–48, 18.0 ± 16.8	QBD (one dose bid) plus CM	HCQ (0.2 g bid) plus PED (20–60 mg/day)	3 months	1) 9) 10) 11) 12)
Wang (2020)	7/30; 25–46, 35.72 ± 7.94	5/32; 23–47, 35.14 ± 7.56	2010 RCMA	2–30, 16.05 ± 4.88	3–28, 15.47 ± 4.79	QBD (one dose bid) plus CM	HCQ (0.1 g bid) plus PED (40 mg qd)	3 months	2) 3) 6) 13) 14) 15)

Abbreviations: ACR, American College of Rheumatology; CM, Chemical medicine of the control group; CTX, cyclophosphamide; HCQ, hydroxychloroquine sulfate; KTR, Kelly’s textbook of rheumatology; NMPA, National Medical Products Administration; PED, prednison or prednisolone acetate; QBD, Qinghao Biejia decoction; RCMA, Rheumatology Branch of Chinese Medical Association; 1), overall response rate; 2), SLEDAI scores; 3), TCM symptoms scores; 4), C-reaction protein; 5), erythrocyte sedimentation rate; 6), C3; 7), Anti-dsDNA antibody; 8), level of lymphocyte subsets in peripheral blood; 9), adverse event(s); 10), IFN-γ; 11), Th1/Th2; 12), IL-4; 13), C4; 14), IgA; 15), IgG; 16), IgM.

### Trial Quality

**FIGURE 2:**
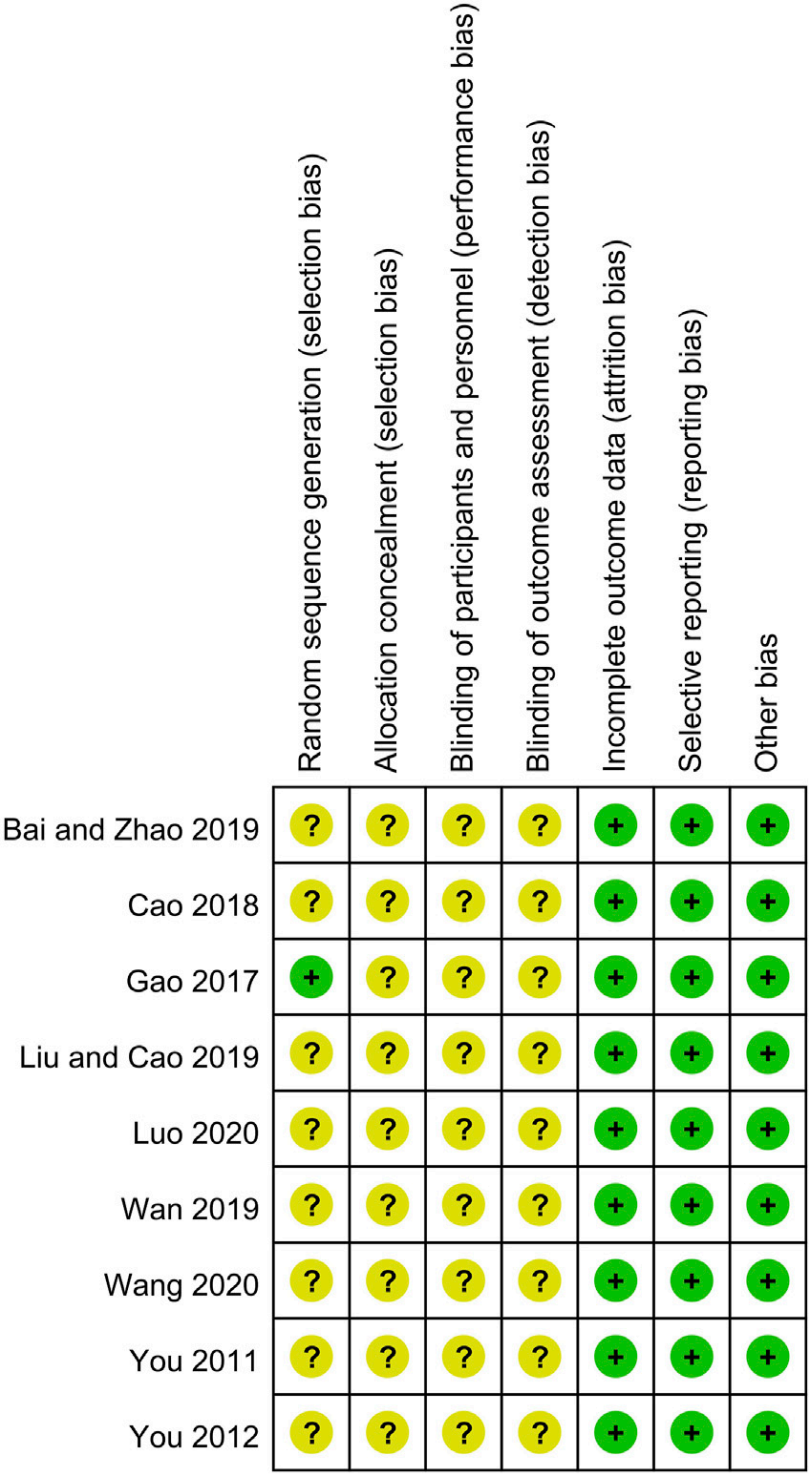
Summary of risk of bias assessment.

The detailed bias assessments of the included trails are depicted in Figure 2. All the nine trials claimed to have used the random allocation method, but only one explicitly pointed out the use of the table of random numbers. Since binding method and allocated procedures were not mentioned in all trials, they were assessed as unclear. The completeness of outcome data and selective reporting of all studies were judged at low risk of bias because no data deficiency and the specified indicators were fully reported. No significant other bias was found in the included studies. None of the above trials provide original information, so we only extract the summary data from the publisher for evaluation, which could makes the analysis possible with information bias.

### Efficacy

#### Overall Response Rate

**FIGURE 3:**
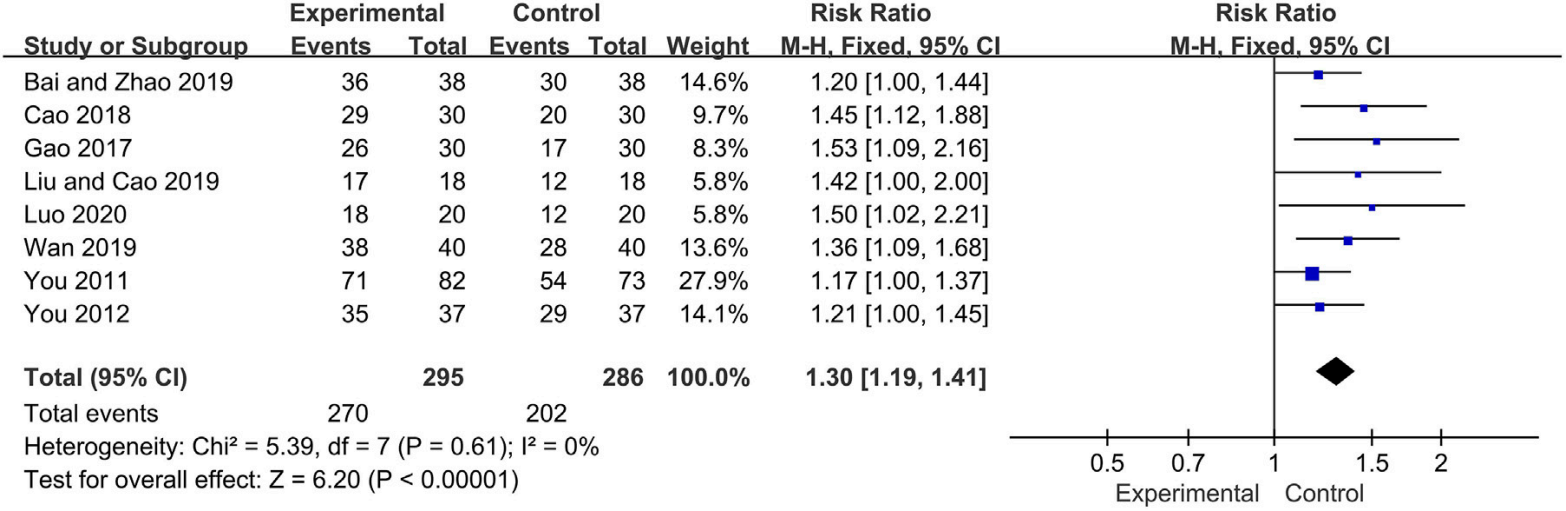
Forest plot of the overall response rate.

Eight trials reported the overall response rate as main outcome. The fixed effect model was used for analysis according to heterogeneity testing (*p* = 0.61, *I* ^*2*^ = 0%). The overall response rate was higher in the experimental group than in control group as a result of treatment (RR = 1.30, 95% CI [1.19, 1.41], *p* < 0.00001) (Figure 3).

#### SLEDAI Scores, TCM Symptoms Scores

**FIGURE 4:**
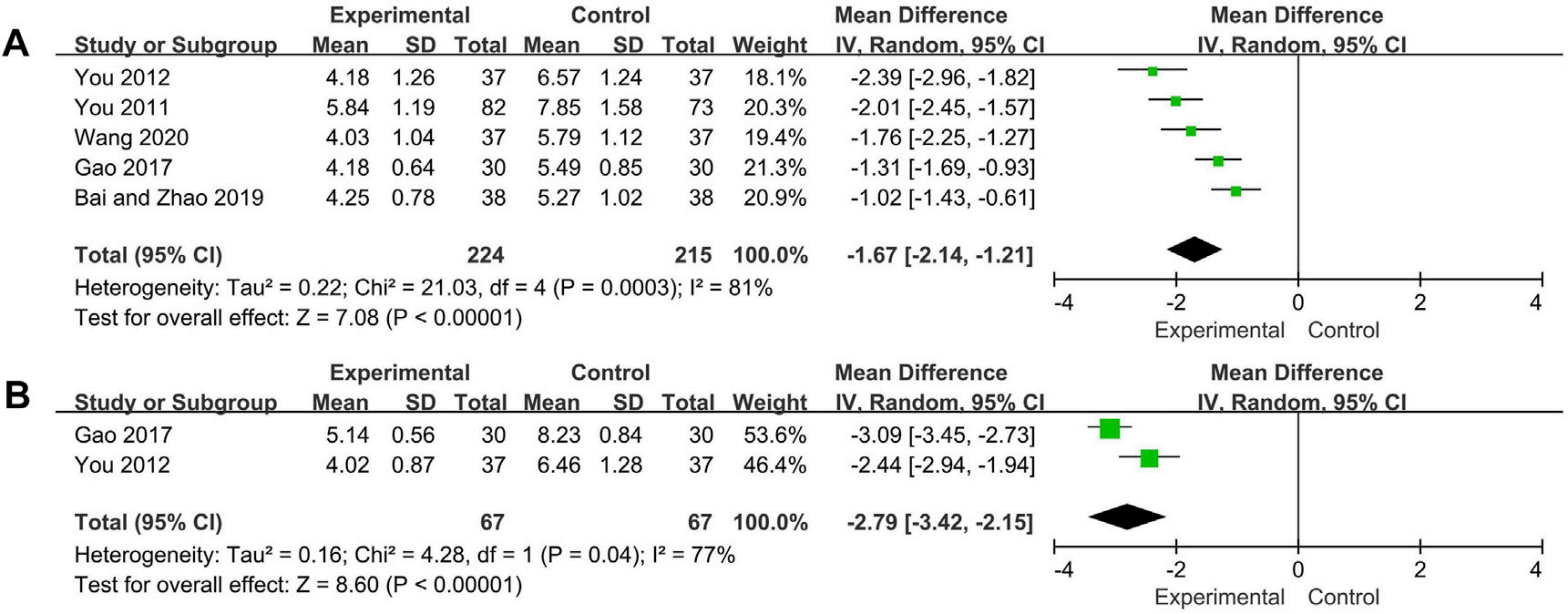
Forest plot of SLEDAI scores **(A)** and TCM symptoms scores **(B)**.

SLEDAI scores of five trials and TCM symptoms scores of two trials were analyzed respectively. The random effect model was used for both analysis due to high heterogeneity was observed (SLEDAI scores: *p* = 0.0003, *I* ^*2*^ = 81%; TCM symptoms scores: *p* = 0.04, *I* ^*2*^ = 77%). The SLEDAI scores and TCM symptoms scores of experimental group were significantly decreased compared with control group (SLEDAI scores: MD = −1.67, 95% CI [−2.14, −1.21], *p* < 0.00001; TCM symptoms scores: MD = −2.79, 95% CI [−3.42, −2.15], *p* < 0.00001) (Figure 4). Two trials (Bai and Zhao, 2019; Wang, 2020) were not included in the TCM symptoms scores analysis because of the use of subdivided TCM symptom scores, both reported results showed that compared with the control group, the experimental group could significantly decreased the TCM symptom scores.

#### C3, C4

**FIGURE 5:**
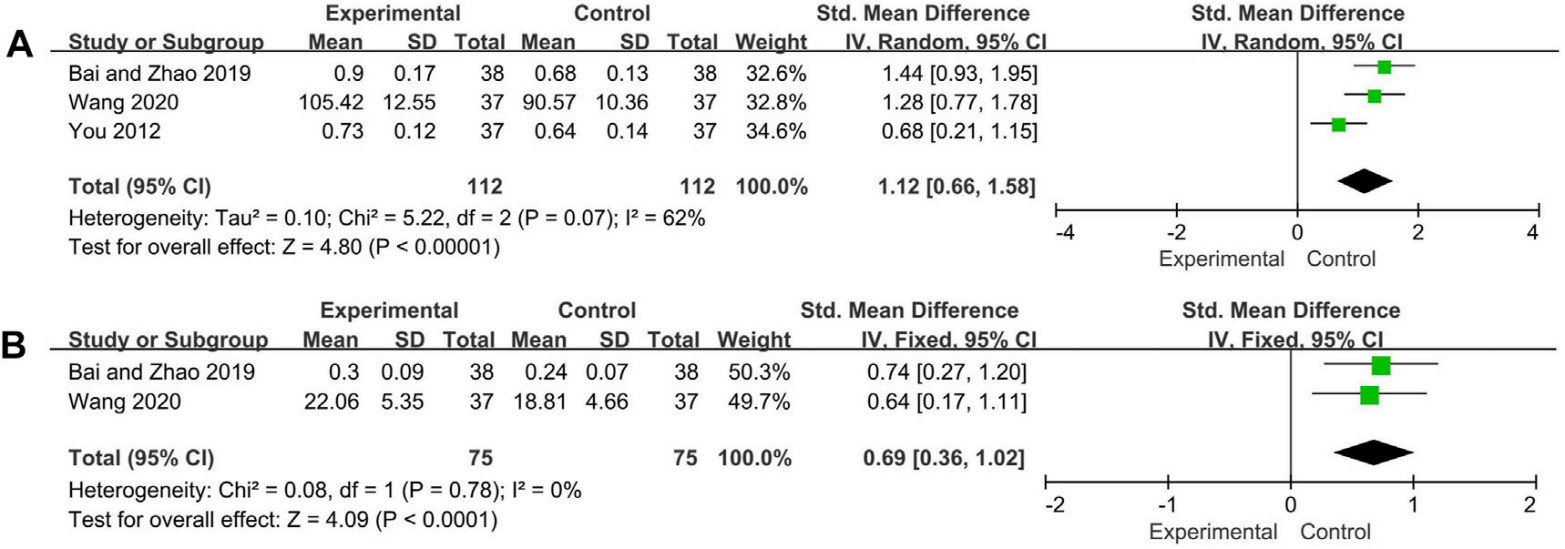
Forest plot of C3 **(A)** and C4 **(B)**.

Three and two trials reported C3 and C4 respectively. The random effect model was adopted for C3 analysis because of its high heterogeneity (*p* = 0.07, *I* ^*2*^ = 62%), and C4 analysis used the fixed effect model (*p* = 0.78, *I* ^*2*^ = 0%). The pooled analysis showed that C3 and C4 of experimental group was significantly increased compared with control group (C3: SMD = 1.12, 95% CI: [0.66, 1.58], *p* < 0.00001; C4: SMD = 0.69, 95%CI: [0.36, 1.02], *p* < 0.0001) (Figure 5).

#### IgG, IgA

**FIGURE 6:**
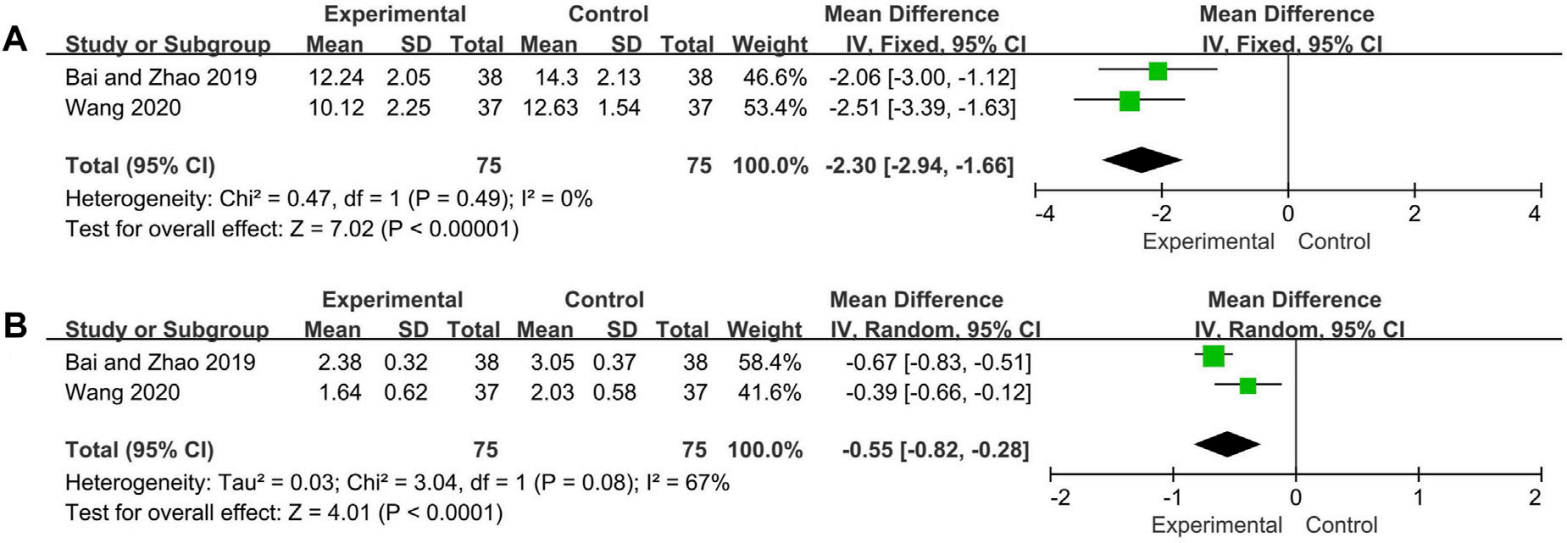
Forest plot of IgG **(A)** and IgA **(B)**.

As per the combined analysis of two included trials, the IgA data were analyzed by the random effect model according to the heterogeneity test (*p* = 0.08, *I* ^*2*^ = 67%), and IgG with low heterogeneity (*p* = 0.49, *I* ^*2*^ = 0%) and hence the fix effect model was utilized. The differences of IgG and IgA between the experimental group and control group were statistically significant (IgG: MD = −2.30, 95% CI [−2.94, −1.66], *p* < 0.00001; IgA: MD = −0.55, 95% CI [−0.82, −0.28], *p* < 0.0001), suggesting the experiment group has better effect in reducing the level of IgG and IgA (Figure 6).

#### IFN-γ, IL-4, Th1/Th2

**FIGURE 7:**
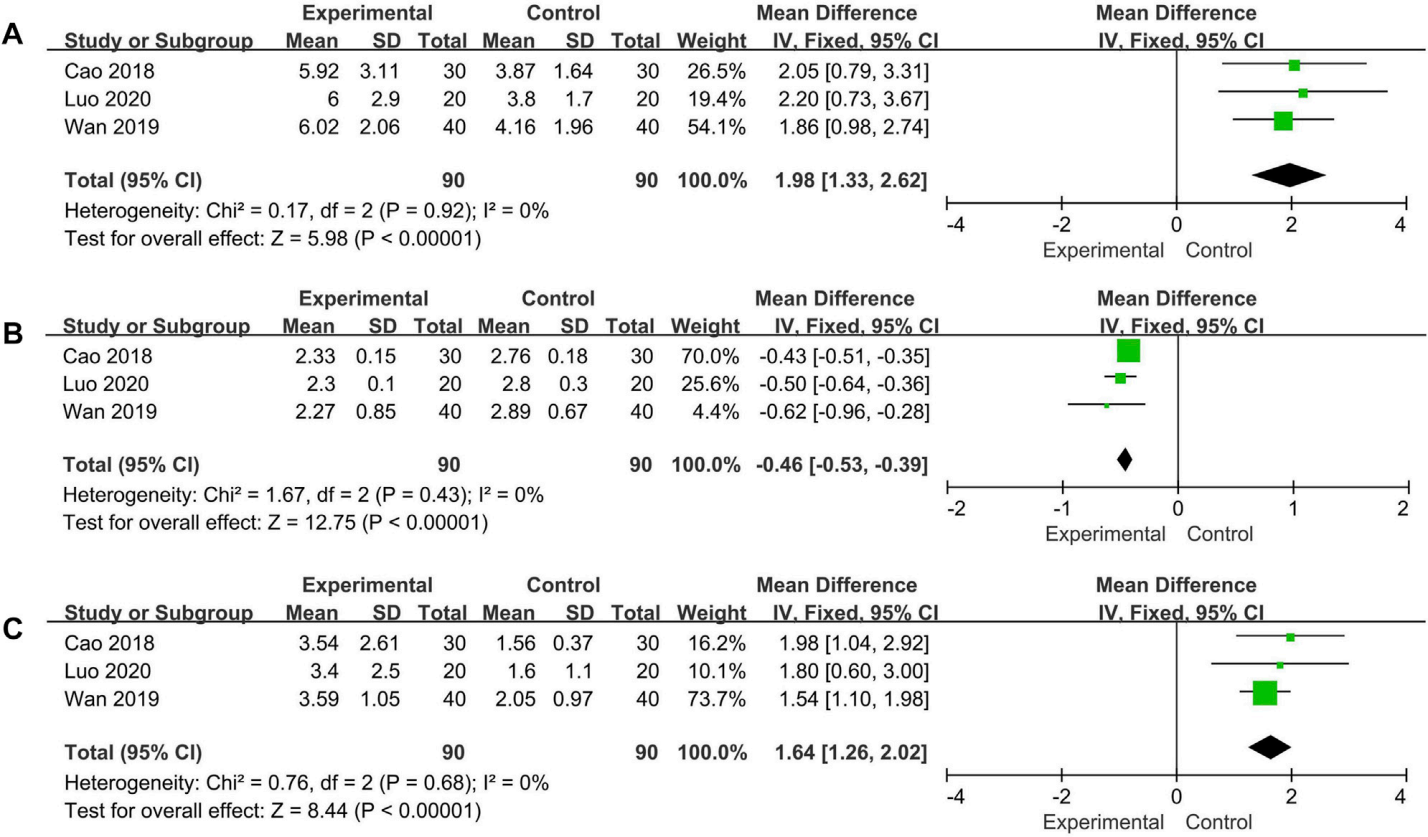
Forest plot of IFN-γ **(A)**, IL-4 **(B)** and Th1/Th2 **(C)**.

Changes of IFN-γ, IL-4, Th1/Th2 were measured in three trials. No significant heterogeneity was noted among the individual trials (IFN-γ: *p* = 0.92, *I* ^*2*^ = 0%; IL-4: *p* = 0.43, *I* ^*2*^ = 0%; Th1/Th2: *p* = 0.68, *I* ^*2*^ = 0%). Using the fixed effect model, the additional use of Qinghao Biejia decoction was found to significantly increased IFN-γ, Th1/Th2 and decreased IL-4 level more than chemical medicine alone (IFN-γ: MD = 1.98, 95% CI: [1.33, 2.62], *p* < 0.00001; IL-4: MD = −0.46, 95% CI: [−0.53, −0.39], *p* < 0.00001; Th1/Th2: MD = 1.64, 95% CI: [1.26, 2.02], *p* < 0.00001) (Figure 7).

### Safety

**FIGURE 8:**
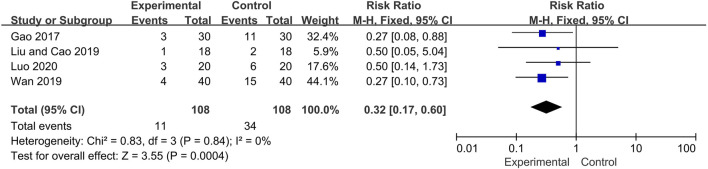
Forest plot of adverse events.

Four included trials described the occurrence of adverse events during the treatment period, including infection (Experiment group: 4 cases; Control group:12 cases), femoral head necrosis (Experiment group: 2 cases; Control group: 7 cases), peptic ulcer (Experiment group: 2 cases; Control group: 9 cases), hypertension (Experiment group: 1 cases; Control group: 2 cases), fundus lesions (Experiment group: 1 cases; Control group: 1 cases), Iatrogenic diabetes (Experiment group: 0 cases; Control group: 1 cases), not specified (Experiment group: 1 cases; Control group: 2 cases). No life-threatening event was reported and no adverse effects of Qinghao Biejia decoction were identified in these trials. Overall, the incidence of adverse events in the experimental group was significantly lower than that in control group (RR = 0.32, 95% CI: [0.17, 0.60], heterogeneity *p* = 0.84, *I* ^*2*^ = 0%) (Figure 8).

### Publication Bias

**FIGURE 9:**
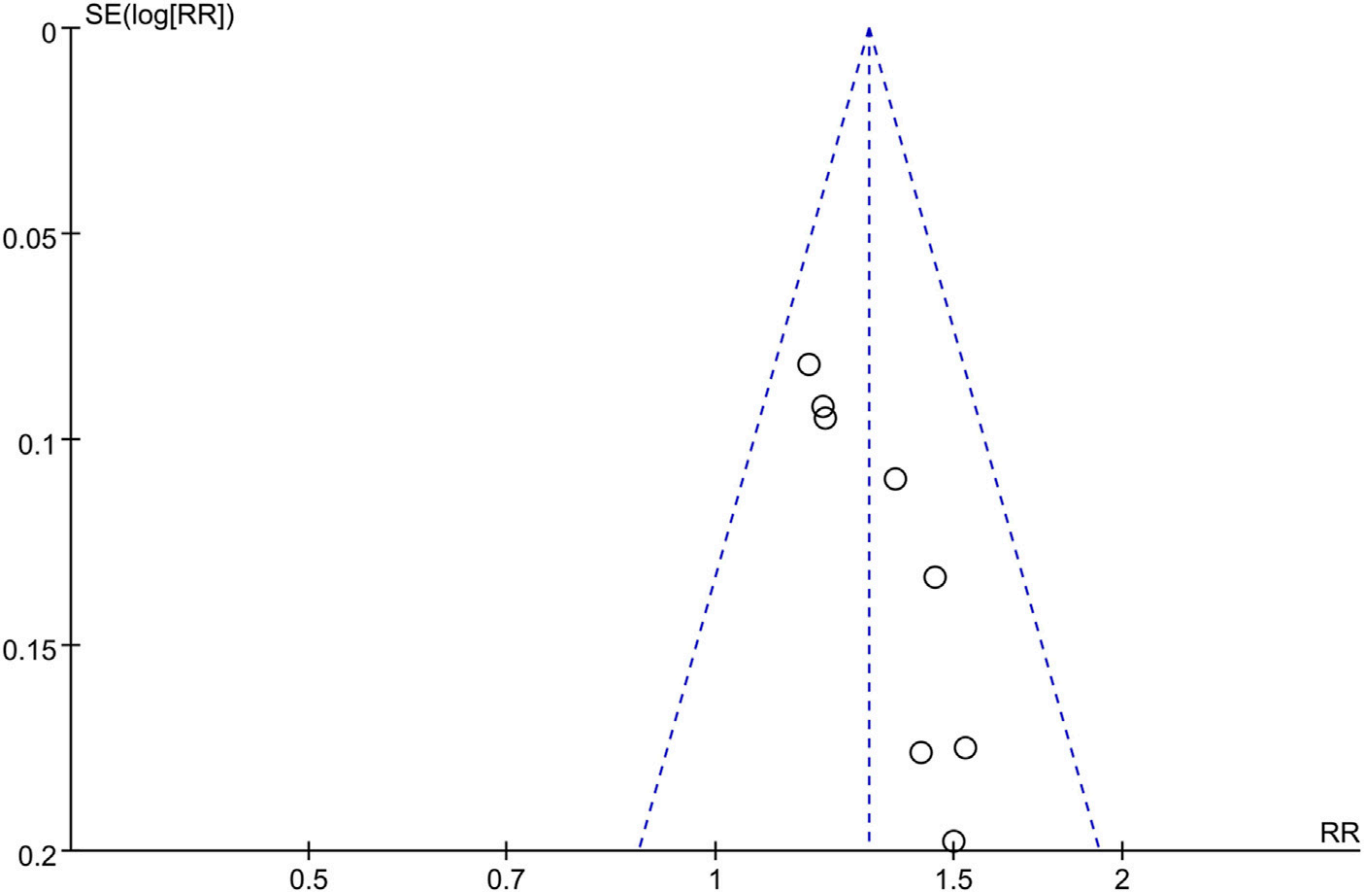
Funnel plot of the overall response rate.

**FIGURE 10:**
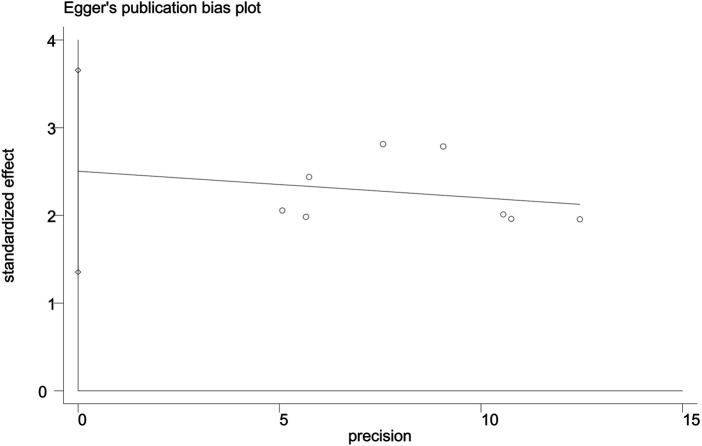
Egger’s test of the overall response rate.

The funnel plot of the overall response rate was asymmetric (Figure 9) and Egger’s test showed *p* = 0.002 (Figure 10), suggesting that there was publication bias in the included studies, which could due to insufficient quantity of included studies and the unpublished negative results.

### Sensitivity Analysis

**FIGURE 11:**
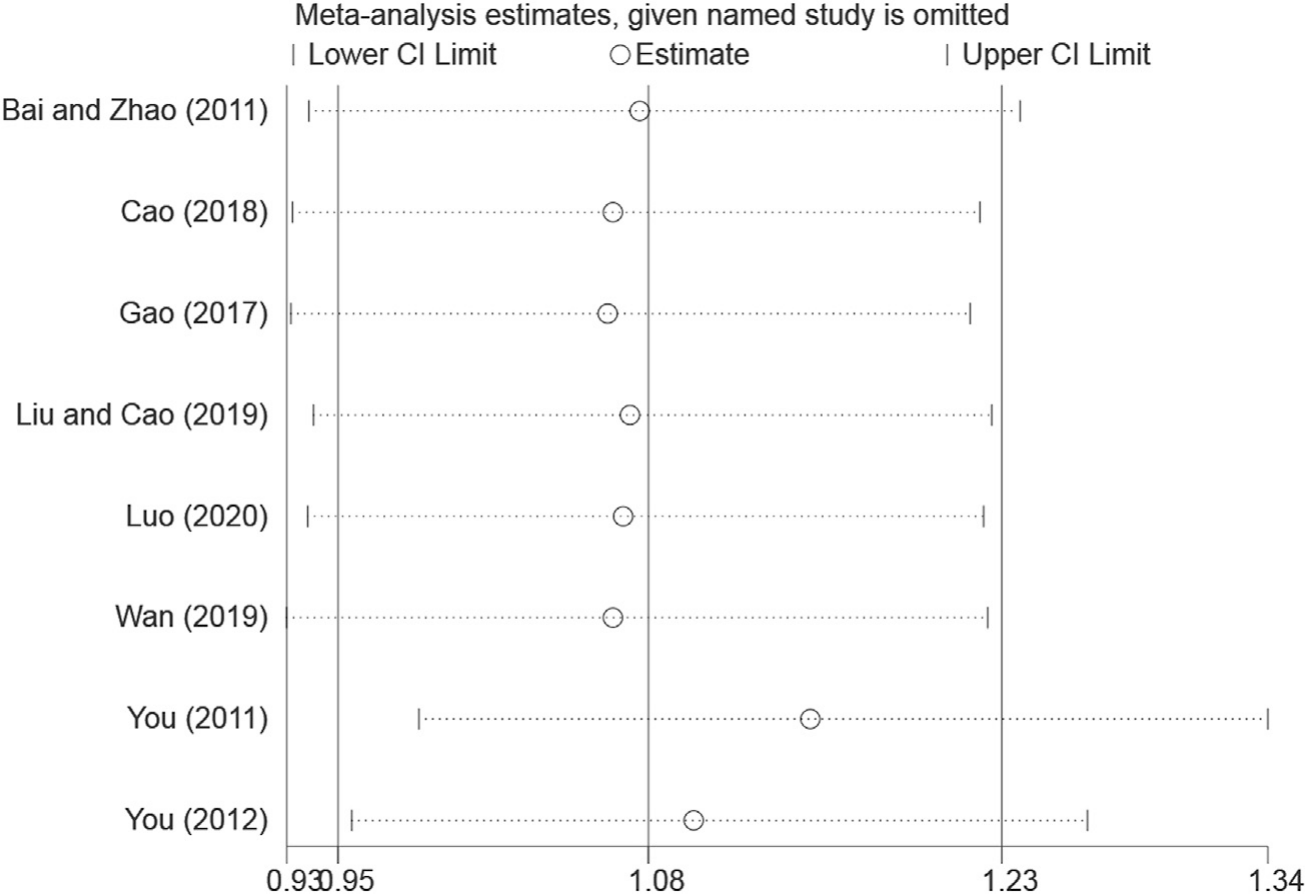
Sensitivity analysis of the overall response rate.

The overall response rate with the largest number of included studies was selected as the index for sensitivity analysis. After the sequential exclusion of each study, re-estimate the RR value of the remaining studies and compared with that before removal to explore whether the meta-analysis results have significant changes or are stable. The overall response rate was found to be similar, with no significant difference (Combined RR = 1.07, 95% CI: [0.95–1.23]) (Table 2, Figure 11).

**TABLE 2:** Sensitivity analysis of total clinical efficacy.

Study ommited	RR	95% CI	
Bai and Zhao (2011)	1.0747516	0.93468136	1.2358127
Cao (2018)	1.0633982	0.92776132	1.218865
Gao (2017)	1.061174	0.92702794	1.2147318
Liu and Cao (2019)	1.0706317	0.93665528	1.2237718
Luo (2020)	1.0677402	0.93418527	1.2203887
Wan (2019)	1.0633923	0.92525262	1.222156
You (2011)	1.1469873	0.98122638	1.3407506
You (2012)	1.097603	0.9528414	1.2643576
Combined	1.0784164	0.9469813	1.2280939

## Discussion

With the improvement of diagnosis and treatment, the survival of lupus patients has improved significantly over the past decades and the 10-years survival rate now exceeds 90%, but patients with SLE still have mortality rates that are two to five times higher than the general population (Bernatsky et al., 2006; Kasitanon et al., 2006). At present, the most common causes of death of SLE patients are renal disease, cardiovascular disease, infection and other complications (Lee et al., 2016). Obviously, duration, dosage and individual susceptibility of steroids and immunosuppressants are factors that cannot be underestimated in the occurrence and development of complications. Traditional Chinese medicine (TCM) plays an important role in the treatment of SLE because of its good efficacy and less toxicity, and can reduce the adverse reactions of chemical medicine. As a result, the treatment of SLE with integrated TCM and chemical medicine has become one of the new research fields.

There is no systemic lupus erythematosus in the Chinese medical classics, but according to its different clinical symptoms, it can be classified as “yin and yang poison”, “butterfly spot”, “sunburn sore”, “blood wind sores”, “warm toxin and spotting” and other categories in Chinese medicine. Contemporary Chinese medicine generally agree that SLE is caused by heat stasis toxin and yin deficiency of kidney (Chen et al., 2019). Yin deficiency and endogenous heat is the core pathogenesis of SLE, blood stasis block throughout the disease, so the treatment principle is nourishing yin and clearing heat, detoxification and removing blood stasis (Han and Jiang, 2017). According to the “Guiding Principles for Clinical Research of New Chinese Medicines” published by the National Medical Products Administration of China in 2002, SLE was divided into blazing heat toxin syndrome, yin deficiency and endogenous heat syndrome, stasis heat arthralgia syndrome, arthralgia caused by wind-damp-heat pathogens syndrome, yang deficiency of spleen and kidney syndrome, yin deficiency of liver and kidney syndrome, deficiency of both qi and blood syndrome, and the TCM symptom inventory (SLEFI) was formulated.

Qinghao Biejia decoction is a common prescription for the treatment of SLE, and also the core prescription for SLE with yin deficiency syndrome (He et al., 2019). It comes from the “Wenbing Tiaobian” by Jutong Wu, a febrile disease expert in Qing Dynasty. In the prescription, Qinghao (*Artemisia annua* L.) and Biejia (*Trionyx sinensis* Wiegmann) are both sovereign medicine, which have the beneficial effects of clearing heat and dispelling pathogenic factor, removing bone steaming and nourishing yin; Zhimu (*Anemarrhena asphodeloides* Bunge) and Shengdihuang (*Rehmannia glutinosa* (Gaertn.) DC.) are both minister drug, which can clear heat-fire, nourish yin for promoting fluid production; Mudanpi (*Paeonia × suffruticosa* Andrews), an assistant medicine, not only clearing heat and cooling blood, but also promoting blood circulation for removing blood stasis. The combination of the above medicines can nourish yin and clear heat, and is suitable for the basic pathogenesis of SLE based on yin deficiency and endogenous heat (Yu, 2012; Lin and Li, 2017). Moreover, the modified Qinghao Biejia decoction enhance the curative effect for SLE through supplementing other traditional Chinese medicine to the original prescription, such as Mohanlian (*Eclipta prostrata* (L.) L.) nourishes liver and kidney, Xuanshen (*Scrophularia ningpoensis* Hemsl.) nourishes yin for descending fire, Rendongteng (*Lonicera japonica* Thunb.) dredges collateral and relieves pain, Baihuasheshecao (*Oldenlandia diffusa* (Willd.) Roxb.), Yinchaihu (*Stellaria dichotoma* var. *lanceolata* Bunge), Digupi (*Lycium chinense* Mill.) and Baiwei (*Cynanchum atratum* Bunge) clear deficient heat and detoxication, Nvzhenzi (*Ligustrum lucidum* W.T.Aiton) tonify and replenish liver and kidney, Gancao (*Glycyrrhiza uralensis* Fisch.) harmonizes all medicines (Wang, 2020). Clinically, the dosages of Qinghao, Biejia, Shengdihuang, Zhimu, Mudanpi are 15, 15, 30, 15, and 20 g, respectively. The preparation method is decocting with water and then taking the supernatant. Take one dose per day, two times in the morning and evening after meals, for 3 or 6 months. Although the dosage of the five TCM components, decoction and administration method in the included trials were the same, there are still some differences. Since six included trials adopted modify Qinghao Biejia decoction, the aforementioned Chinese medicines were added according to the patient’s condition, the medication were not completely consistent contributed to certain heterogeneity in our analysis. Qinghao Biejia decoction prepared in the included trials lacked a standardization due to the amount of water added and supernatant were not uniform, as well as temperature and dryness of extract were not clarified. This is the deficiency of Chinese medicine decoction in clinical application. In the future, it could be prepared into granules, capsules and other formulations for standardized use.

Qinghao Biejia decoction, as a traditional Chinese medicine compound decoction, has complex chemical components, and the major bioactive compounds for treating SLE may include the following. Artemisinin, an effective component of Qinghao, has potential beneficial effects for SLE include improving symptoms, reducing level of antibodies and proteinuria, ameliorating renal damage (Mu and Wang, 2018). Biejia contains trionyx sinensis polysaccharides, collagen, amino acids and trace elements, etc., which have anti-fatigue and promote immune function, and can inhibit connective tissue proliferation and increase plasma protein (Wang et al., 2016). The iridoid glycosides in Shengdihuang can enhance immune function and anti inflammation (Chen et al., 2021). The mangiferin and total polysaccharides in Zhimu have significant anti-inflammatory and antipyretic effects (Weng et al., 2018). Paeonol, paeoniflorin and other glycosides in Mudanpi have anti-inflammatory effects and can treat immune dysfunction in patients with SLE (Wang and Chen, 2015). A large number of experimental and clinical researches have shown that Qinghao Biejia decoction can reduce fever, anti inflammatory, improve immunity, etc., and has obvious therapeutic effects on SLE (Xia and Chen, 2019). Lin et al. (Lin et al., 2014, Lin et al., 2019) reported that inhibiting the expression of Th17 cells and IL-17, improving the pathological changes in renal tissues may be one of the mechanisms of Qinghaobiejia decoction in treating SLE.

Because SLE has a high degree of heterogeneity, complex pathophysiology and multiple clinical manifestations, no single test is sensitive or specific enough to diagnose. Protein biomarkers, including certain autoantibodies, complements and cytokines, play essential roles in development of SLE. The American College of Rheumatology SLE criteria place emphasis on the diagnostic and prognostic values of protein biomarker. Anti-double-stranded (anti-dsDNA) antibodies are the canonical parameters in SLE patients. They can directly target annexin II and alpha-actinin expressed in mesangial cells and proximal renal tubular epithelial cells, or indirectly bind to constituents of the GBM to induce increased secretion of proinflammatory cytokines (Qi et al., 2018). Only one included trial (You, 2012) determined anti-dsDNA antibodies, so we did not perform statistical analysis. However, the results of this trial indicated the additional use of Qinghao Biejia decoction has a better effect on trans-negative rate of anti-dsDNA antibodies than chemical medicine alone (number of positive antibody cases before and after treatment: experimental group 15/30, control group 24/32). SLE patients generally have obvious abnormal cellular and humoral immune functions, such as increased serum immunoglobulins (IgA, IgG, IgM) and decreased complement (C3, C4) levels. The imbalance of cytokine (Th1/Th2) also occurs and can determine disease activity (Jung et al., 2013; Talaat et al., 2015; Guimaraes et al., 2017; Qi et al., 2018). Therefore, enhancing the detection of the above indicators is helpful to understand the diseases activity of SLE and evaluate the clinical efficacy. Based on this meta-analysis, the additional use of Qinghao Biejia decoction is not only superior to chemical medicine alone in people with SLE in terms of improving the overall response rate, reducing SLEDAI and TCM symptom scores, but also significantly improving immunological indicators, manifested in IgG and IgA decreased, C3 and C4 levels increased, cytokine balance restored. Thus it can be considered that Qinghao Biejia decoction has definite curative effect on SLE and can improve the immune function of SLE patients. And the adverse events was less, suggesting that the combination of Qinghao Biejia decoction can reduce the occurrence of adverse reactions in clinical applications.

Nevertheless, our research has several limitations as follows: 1) The participants in the included studies are all from China, which may have ethnic and regional differences, and the course of disease, age and intervention methods of each included study population are not completely consistent; 2) Treatment duration and outcome indicators are not the same, prone to selection bias; 3) All the included studies are Chinese document, which may have language bias. 4) Only four included studies reported the occurrence of adverse events, and safety monitoring may not be sufficient. 5) The research has publication bias due to insufficient quantity of included studies. 6) Subgroup analysis was not performed according to the course of treatment, medication and dosage. Overall, the conclusions of our research need to be further verified by well-designed, stringently enforced, large-sample clinical trials.

## Conclusion

This meta-analysis demonstrated that the additional use of Qinghao Biejia decoction has more advantages in the treatment of SLE than conventional chemical medication alone, which could enhance the efficacy and reduce adverse reactions, and is worthy of clinical promotion. However, due to the limitations of the included studies, high-quality trials with well-designed, stringently enforced, and large number of samples are required to further confirm the long-term efficacy and safety of Qinghao Biejia decoction.

## Data Availability

The original contributions presented in the study are included in the article/Supplementary Material, further inquiries can be directed to the corresponding authors.
