# Supplementary material for: Efficacy and Safety of Qinghao Biejia Decoction in the Treatment of Systemic Lupus Erythematosus: A Systematic Review and Meta-Analysis
Source: Front Pharmacol. 2021 Aug 6;12:669269. doi: 10.3389/fphar.2021.669269 (PMC8378134; doi:10.3389/fphar.2021.669269)
Supplement: Supplementary file 1 [file Table1.DOC]

Supplementary Table 1 Patented formulations and chemical

| **Study** | **Formulation** | **Source** | **Species, concentration** | **Quality control reported? (Y/****N)** | **The most representative active components of the plants in the treatment of SLE** |
| --- | --- | --- | --- | --- | --- |
| You (2011) | Prednison acetate | Not specified | Not specified | N | 1. Qinghao: artemisinin (improve symptoms, reduce level of antibodies and proteinuria, ameliorate renal damage); 2. Biejia: trionyx sinensis polysaccharides, collagen, amino acids and trace elements (anti fatigue and promote immune function, inhibit connective tissue proliferation and increase plasma protein); 3. Zhimu: mangiferin and total polysaccharides (anti inflammation, antipyretic); 4. Shengdihuang: iridoid glycosides (enhance immune function and anti inflammation); 5. Mudanpi: paeonol, paeoniflorin and other glycosides (anti inflammation and regulate immunity); |
|  | Qinghao Biejia decoction | Prepared by You (2011) | 1. Qinghao: aerial part of *Artemisia annua* L., 15 g 2. Biejia: carapace of Trionyx sinensis Wiegmann, 15 g 3. Zhimu: rhizome of *Anemarrhena asphodeloides* Bunge, 15 g 4. Shengdihuang: root of *Rehmannia glutinosa* (Gaertn.) DC., 30 g 5. Mudanpi: root bark of *Paeonia × suffruticosa* Andrews, 20 g | N - Decoct with water |  |
| You (2012) | Prednison acetate | Not specified | Not specified | N |  |
|  | Cyclophosphamide | Not specified | Not specified | N |  |
|  | Modify Qinghao Biejia decoction | Prepared by You (2012) | 1. Qinghao:aerial part of *Artemisia annua* L., 15 g 2. Biejia: carapace of Trionyx sinensis Wiegmann, 15 g 3. Zhimu: rhizome of *Anemarrhena asphodeloides* Bunge, 15 g 4. Shengdihuang: root of *Rehmannia glutinosa* (Gaertn.) DC., 30 g 5. Mudanpi: root bark of *Paeonia × suffruticosa* Andrews, 20 g 6. Mohanlian: aerial part of *Eclipta prostrata* (L.) L., 20 g 7. Nvzhenzi: mature fruit of *Ligustrum lucidum* W.T.Aiton, 20 g 8. Xuanshen: root of *Scrophularia ningpoensis* Hemsl., 20 g 9. Maidong: root of *Ophiopogon japonicus* (Thunb.) Ker Gawl., 20 g 10. Yinchaihu: root of *Stellaria dichotoma* var. *lanceolata* Bunge, 15 g 11. Baiwei: root and rhizome of *Cynanchum atratum* Bunge, 15 g 12. Digupi: root bark of *Lycium chinense* Mill., 15 g 13. Baihuasheshecao: aerial part of *Oldenlandia diffusa* (Willd.) Roxb., 30 g 14. Rendongteng: stem and branch of *Lonicera japonica* Thunb., 30 g | N - Decoct with water |  |

Supplementary Table 1 (continue)

| **Study** | **Formulation** | **Source** | **Species, concentration** | **Quality control reported? (****Y/N)** | **The most representative active components of the plants in the treatment of SLE** |
| --- | --- | --- | --- | --- | --- |
| Gao et al. (2017) | Hydroxychloroquine sulfate | Shanghai Zhongxi Pharmaceutical Co.,Ltd. | Specification: 0.1 g | Y - National medicine permission number: H19990263 | 1. Mohanlian: luteolin and quercetin (regulate immunity), wedelolactone (anti inflammation and anti apoptosis); 2. Nvzhenzi: salidroside and polysaccharides (regulate immunity), nuezhenoside (anti inflammation); 3. Xuanshen: polysaccharides (anti fatigue), iridoid glycosides (anti oxidant), nuezhenoside (analgesic); 4. Maidong: furostan saponin A, furostan saponin B and total saponins (anti inflammation), polysaccharides (regulate immunity); 5. Yinchaihu: paeonol and alkaloid (anti inflammation and antipyretic); 6. Baiwei: C21 steroidal glycosides (suppress immunity), Cynatratoside C (anti inflammation); |
|  | Prednisolone acetate | Shandong Xinhua Pharmaceutical Co.,Ltd. | Specification: 5 mg | Y - National medicine permission number: H37020648 |  |
|  | Modify Qinghao Biejia decoction | Prepared by Gao  et al. (2017) | 1. Qinghao: aerial part of *Artemisia annua* L., 15 g 2. Biejia: carapace of Trionyx sinensis Wiegmann, 15 g 3. Zhimu: rhizome of *Anemarrhena asphodeloides* Bunge, 15 g 4. Shengdihuang: root of *Rehmannia glutinosa* (Gaertn.) DC., 30 g 5. Mudanpi: root bark of *Paeonia × suffruticosa* Andrews, 20 g 6. Mohanlian: aerial part of Eclipta prostrata (L.) L., 20 g 7. Nvzhenzi: mature fruit of Ligustrum lucidum W.T.Aiton, 15 g 8. Xuanshen: root of Scrophularia ningpoensis Hemsl., 20 g 9. Yinchaihu: root of Stellaria dichotoma var. lanceolata Bunge, 15 g 10. Baiwei: root and rhizome of Cynanchum atratum Bunge, 15 g 11. Digupi: root bark of Lycium chinense Mill., 15 g 12. Baihuasheshecao: aerial part of Oldenlandia diffusa (Willd.) Roxb., 30 g 13. Rendongteng: stem and branch of Lonicera japonica Thunb., 30 g | N - add 800 mL water to decoct and filter, then take 150 mL supernatant |  |

Supplementary Table 1 (continue)

| **Study** | **Formulation** | **Source** | **Species, concentration** | **Quality control reported? (Y/N)** | **The most representative active components of the plants in the treatment of SLE** |
| --- | --- | --- | --- | --- | --- |
| Cao (2018) | Hydroxychloroquine sulfate | Shanghai Zhongxi Pharmaceutical Co.,Ltd. | Not specified | Y - National medicine permission number: H19990263 | 1. Digupi: scopoletin and scopolin (analgesic and anti inflammation), flavones and anthraquinones (treat skin diseases and regulate immunity); 2. Baihuasheshecao: kaempferol, quercetin, caffeic acid, rutin, ursolic acid, oleanolic acid, geniposide, 2-methyl-3-hydroxyanthraquinone, emodin, p-coumaric acid, ferulic acid, stigmasterol (enhance immunity, anti inflammation and anti oxidant); 3. Rendongteng: chlorogenic acid, caffeic acid, hedera sapogenin, loniceraflavone, luteolin (anti inflammation, antipyretic, regulate immunity); 4. Gancao: glycyrrhizin, isoliquiritigenin, polysaccharides (anti inflammation, regulate immunity). |
|  | Cyclophosphamide | Zhejiang Hisun Pharmaceutical Co.,Ltd. | Not specified | Y - National medicine permission number: H20084627 |  |
|  | Qinghao Biejia decoction | The Chinese herbal purchased from Suzhou Tianling Chinese Herbal Medicine Co.Ltd.  The formulation was prepared by Cao (2018) | 1. Qinghao: aerial part of *Artemisia annua* L., 15 g 2. Biejia: carapace of Trionyx sinensis Wiegmann, 15 g 3. Zhimu: rhizome of *Anemarrhena asphodeloides* Bunge, 15 g 4. Shengdihuang: root of *Rehmannia glutinosa* (Gaertn.) DC., 30 g 5. Mudanpi: root bark of *Paeonia × suffruticosa* Andrews, 20 g | N |  |
| Liu and Cao (2019) | Hydroxychloroquine sulfate | Shanghai Zhongxi Pharmaceutical Co.,Ltd. | Not specified | Y-national medicine permission number: H19990263 |  |
|  | Cyclophosphamide | Hanhui Pharmaceutical Co.,Ltd. | Not specified | Y-national medicine permission number: H20093392 |  |
|  | Qinghao Biejia decoction | Prepared by Liu and Cao (2019) | 1. Qinghao: aerial part of *Artemisia annua* L., 15 g 2. Biejia: carapace of Trionyx sinensis Wiegmann, 15 g 3. Zhimu: rhizome of *Anemarrhena asphodeloides* Bunge, 15 g 4. Shengdihuang: root of *Rehmannia glutinosa* (Gaertn.) DC., 30 g 5. Mudanpi: root bark of *Paeonia × suffruticosa* Andrews, 20 g | N |  |

Supplementary Table 1 (continue)

| **Study** | **Formulation** | **Source** | **Species, concentration** | **Quality control reported? (Y/N)** |  |
| --- | --- | --- | --- | --- | --- |
| Bai and Zhao (2019) | Hydroxychloroquine sulfate | Not specified | Not specified | N |  |
|  | Prednisolone acetate | Not specified | Not specified | N |  |
|  | Modify Qinghao Biejia decoction | Prepared by Bai and Zhao (2019) | 1. Qinghao: aerial part of *Artemisia annua* L., 15 g 2. Biejia: carapace of Trionyx sinensis Wiegmann, 15 g 3. Zhimu: rhizome of *Anemarrhena asphodeloides* Bunge, 15 g 4. Shengdihuang: root of *Rehmannia glutinosa* (Gaertn.) DC., 30 g 5. Mudanpi: root bark of *Paeonia × suffruticosa* Andrews, 20 g 6. Mohanlian: aerial part of Eclipta prostrata (L.) L., 20 g 7. Nvzhenzi: mature fruit of Ligustrum lucidum W.T.Aiton, 15 g 8. Xuanshen: root of Scrophularia ningpoensis Hemsl., 20 g 9. Yinchaihu: root of Stellaria dichotoma var. lanceolata Bunge, 15 g 10. Baiwei: root and rhizome of Cynanchum atratum Bunge, 15 g 11. Digupi: root bark of Lycium chinense Mill., 15 g 12. Baihuasheshecao: aerial part of Oldenlandia diffusa (Willd.) Roxb., 30 g 13. Rendongteng: stem and branch of Lonicera japonica Thunb., 30 g | N - add 700 mL water to decoct, then take 200 mL supernatant |  |

Supplementary Table 1 (continue)

| **Study** | **Formulation** | **Source** | **Species, concentration** | **Quality control reported? (Y/N)** |  |
| --- | --- | --- | --- | --- | --- |
| Wan (2019) | Hydroxychloroquine sulfate | Not specified | Not specified | N |  |
|  | Prednisolone acetate | Not specified | Not specified | N |  |
|  | Modify Qinghao Biejia decoction | Prepared by Wan (2019) | 1. Qinghao: aerial part of *Artemisia annua* L., 15 g 2. Biejia: carapace of Trionyx sinensis Wiegmann, 15 g 3. Zhimu: rhizome of *Anemarrhena asphodeloides* Bunge, 15 g 4. Shengdihuang: root of *Rehmannia glutinosa* (Gaertn.) DC., 30 g 5. Mudanpi: root bark of *Paeonia × suffruticosa* Andrews, 20 g 6. Mohanlian: aerial part of Eclipta prostrata (L.) L., 20 g 7. Nvzhenzi: mature fruit of Ligustrum lucidum W.T.Aiton, 15 g 8. Xuanshen: root of Scrophularia ningpoensis Hemsl., 20 g 9. Yinchaihu: root of Stellaria dichotoma var. lanceolata Bunge, 15 g 10. Baiwei: root and rhizome of Cynanchum atratum Bunge, 15 g 11. Digupi: root bark of Lycium chinense Mill., 15 g 12. Baihuasheshecao: aerial part of Oldenlandia diffusa (Willd.) Roxb., 30 g 13. Rendongteng: stem and branch of Lonicera japonica Thunb., 30 g | N - add 800 mL water to decoct and filter, then take 150 mL supernatant |  |

Supplementary Table 1 (continue)

| **Study** | **Formulation** | **Source** | **Species, concentration** | **Quality control reported? (Y/N)** |  |
| --- | --- | --- | --- | --- | --- |
| Luo (2020) | Hydroxychloroquine sulfate | Not specified | Not specified | N |  |
|  | Prednison acetate | Not specified | Not specified | N |  |
|  | Modify Qinghao Biejia decoction | Prepared by Luo (2020) | 1. Qinghao: aerial part of *Artemisia annua* L., 15 g 2. Biejia: carapace of Trionyx sinensis Wiegmann, 15 g 3. Zhimu: rhizome of *Anemarrhena asphodeloides* Bunge, 15 g 4. Shengdihuang: root of *Rehmannia glutinosa* (Gaertn.) DC., 30 g 5. Mudanpi: root bark of *Paeonia × suffruticosa* Andrews, 20 g 6. Mohanlian: aerial part of Eclipta prostrata (L.) L., 20 g 7. Nvzhenzi: mature fruit of Ligustrum lucidum W.T.Aiton, 15 g 8. Xuanshen: root of Scrophularia ningpoensis Hemsl., 20 g 9. Yinchaihu: root of Stellaria dichotoma var. lanceolata Bunge, 15 g 10. Baiwei: root and rhizome of Cynanchum atratum Bunge, 15 g 11. Digupi: root bark of Lycium chinense Mill., 15 g 12. Baihuasheshecao: aerial part of Oldenlandia diffusa (Willd.) Roxb., 30 g 13. Rendongteng: stem and branch of Lonicera japonica Thunb., 30 g | N - add water to decoct, then take 150 mL supernatant |  |

Supplementary Table 1 (continue)

| **Study** | **Formulation** | **Source** | **Species, concentration** | **Quality control reported? (Y/N)** |  |
| --- | --- | --- | --- | --- | --- |
| Wang (2020) | Prednison acetate | Jiangxi Xi'er Kangtai Pharmaceutical Co.,Ltd. | Specification: 40 mg | Y - National medicine permission number: H36021023 |  |
|  | Hydroxychloroquine sulfate | Shanghai Zhongxi Pharmaceutical Co.,Ltd. | Specification: 0.1 g | Y - National medicine permission number: H19990263 |  |
|  | Modify Qinghao Biejia decoction | Prepared by Wang (2020) | 1. Qinghao: aerial part of *Artemisia annua* L., 15 g 2. Biejia: carapace of Trionyx sinensis Wiegmann, 15 g 3. Zhimu: rhizome of *Anemarrhena asphodeloides* Bunge, 15 g 4. Shengdihuang: root of *Rehmannia glutinosa* (Gaertn.) DC., 30 g 5. Mudanpi: root bark of *Paeonia × suffruticosa* Andrews, 20 g 6. Mohanlian: aerial part of Eclipta prostrata (L.) L., 20 g 7. Nvzhenzi: mature fruit of Ligustrum lucidum W.T.Aiton, 15 g 8. Xuanshen: root of Scrophularia ningpoensis Hemsl., 20 g 9. Yinchaihu: root of Stellaria dichotoma var. lanceolata Bunge, 15 g 10. Baiwei: root and rhizome of Cynanchum atratum Bunge, 15 g 11. Digupi: root bark of Lycium chinense Mill., 15 g 12. Baihuasheshecao: aerial part of Oldenlandia diffusa (Willd.) Roxb., 30 g 13. Rendongteng: stem and branch of Lonicera japonica Thunb., 30 g 14. Gancao: root and rhizome of *Glycyrrhiza uralensis* Fisch., 15 g | N - Add 800mL water to soak for 0.5 h, and decoct to 150 mL |  |
